# Reliability and validity of the Spinal Appearance Questionnaire (SAQ) and the Trunk Appearance Perception Scale (TAPS)

**DOI:** 10.1186/s13018-018-0980-1

**Published:** 2018-10-30

**Authors:** Meinald T. Thielsch, Mark Wetterkamp, Patrick Boertz, Georg Gosheger, Tobias L. Schulte

**Affiliations:** 1 Department of Psychology, University of Münster, Fliednerstr. 21, 48149 Münster, Germany; 2 St. Josef-Hospital, Ruhr University Bochum, Bochum, Germany; 3 Universitätsklinikum Münster, Münster, Germany

**Keywords:** Idiopathic scoliosis, Body image, Assessment, Psychometric properties, Validation

## Abstract

**Background:**

The Spinal Appearance Questionnaire (SAQ) and the Trunk Appearance Perception Scale (TAPS) are questionnaires that mostly rely on drawings to assess scoliosis patients’ subjective viewpoints on their trunk deformity. Our aim was to perform an in-depth assessment of the psychometric quality of both measures, the SAQ (version 1.1) and TAPS, and compare them to provide practical recommendations.

**Methods:**

Web-based survey study with 255 patients suffering from idiopathic scoliosis (age 30.0 ± 16.7 years, Cobb angle 43.5 ± 20.9°) and 189 matched healthy control individuals. Participants answered a broad set of validated questionnaires including SRS 22-r, PHQ-9, PANAS, FKS, WHO-5, BFI-S, and PTQ. We calculated reliability (Cronbach’s *α*, test–retest correlations) as well as factorial, convergent, divergent, concurrent, and discriminant validity.

**Results:**

Reliability was high (Cronbach’s *α* ≥ .86; test–retest *r* ≥ .80), except for test–retest correlation of the SAQ Expectations scale (*r* = 0.67). Both the SAQ and TAPS measures showed clear factor solutions, indicating factorial validity. High correlations with theoretically related measures (e.g., SRS 22-r, overall stress, Cobb angle) indicated convergent validity. Moderate correlations occurred with concurrent criteria such as mood, depression, body dysmorphic disorder, and well-being. The matched-pair analysis revealed strong evidence for discriminant validity (Cohen’s *d* > 2 for SAQ total score and TAPS). Subgroup analyses showed that patients with more severe Cobb angles (≥ 40°) and those ≥ 46 years of age had significantly worse SAQ and TAPS scores.

**Conclusion:**

We recommend using the TAPS for future clinical workups and research, as it is much shorter and revealed slightly higher psychometric quality in comparison to the SAQ.

**Electronic supplementary material:**

The online version of this article (10.1186/s13018-018-0980-1) contains supplementary material, which is available to authorized users.

## Background

In recent years, specific scales for the in-depth evaluation of scoliosis patients’ subjective viewpoints on their trunk deformity have been developed [1–3]. Most of such scales use questions in the form of statements, yet two specific instruments encompass drawings: The Spinal Appearance Questionnaire (SAQ) and the Trunk Appearance Perception Scale (TAPS), both originating from the Walter Reed Visual Assessment Scale (WRVAS) [4–6].

The WRVAS focuses on patients’ appearances, but it fails to ask about patients’ satisfaction with their body image [6]. Also, as reported by Bago et al., some of the WRVAS drawings do not directly correlate with the equivalent radiological deformity, and adolescents can have difficulties in comprehending the questionnaire [5, 7]. Therefore, Sanders et al. created the SAQ, which was further modified by Carreon et al. to address these specific limitations [4, 8]. This current modified version of the SAQ, the SAQ v1.1, is the focus of the present study; for readability, we will refer to it only as SAQ (meaning SAQ v.1.1) in the following text. This questionnaire consists of 11 pictorial items and 22 questions regarding patients’ expectations. Yet, based on data from 1802 patients, Carreon et al. found that only 14 of the items loaded on two factors: The first ten drawings on the so-called Appearance factor and four questions (#12–15) on an Expectations factor. Thus, the authors recommended using only these 14 items. The authors also reported evidence for good reliability (Cronbach’s *α* ≥ 0.88; test–retest correlation ≥ 0.81) and convergent validity of the SAQ in terms of correlations with the major curve magnitude (0.324 ≤ *r* ≤ 0.361, *P* < 0.01), and they argued for discriminant validity by showing significant differences between patients receiving different treatments [8]. Yet, they have not yet performed a comparison with healthy controls or a systematic analysis of further convergent criteria (especially psychological criteria and patients’ well-being). Furthermore, Mulcahey et al. [7] found that a large percentage of younger patients (between 8 and 16 years old) had difficulties understanding items and illustrations in the child version of the SAQ. Finally, there is an imbalance between the SAQ Appearance and the SAQ Expectation scales, as the Appearance scale makes up about 70% of the total SAQ score. Thus, in psychometric analyses, both subscales should be examined in detail and separately.

The other questionnaire of interest in this study, the TAPS, was created by Bago et al. [5]. It consists of only three drawings illustrating the patient’s trunk from three different angles: First, looking at the back of the patient in an upright position; second, looking at the front of the patient from their head towards the pelvis while the patient is bent over towards the observer; and third, looking at the front of the patient in an upright position (this third drawing has a version for females and a version for males) [9]. The authors tested 186 patients and found evidence for good to excellent reliability (Cronbach’s *α* = 0.89; test–retest correlation for *n* = 35 patients was 0.92). Furthermore, Bago et al. reported convergent validity in terms of high correlations with the SRS-22 and discriminant validity by finding high correlations between TAPS and the largest curve in terms of Cobb angle (CMAX) [5]. In additional studies, Misterka et al. reported high correlations between TAPS and the main Cobb angle (*r* = − 0.44, *P* < 0.05, *n* = 36) [10]; Rigo et al. reported high correlations between TAPS and self-image and pain scales in the SRS-22 (*n* = 71) [11]. Nonetheless, currently, the TAPS has only been assessed with relatively small samples; it is still missing a factor analysis, a comparison to healthy controls, and further systematic analysis of additional convergent criteria.

Matamalas et al. was the first to directly compare the SAQ with the TAPS based on a sample of 80 patients (with Cobb angles ≥ 25°, mean age 20.3 years). They found nearly identical reliability values (in terms of Cronbach’s *α*) as the original studies, high correlation with the SRS-22 and with radiological magnitude of the curve, and a correlation of *r* = − 0.80 between the SAQ Appearance scale and TAPS. Matamalas et al. favored the TAPS over the SAQ because it is shorter [12]. Still, to validly compare the SAQ and the TAPS, there needs to be a broad prospective cohort study that (a) examines patients of different ages with a wide range of Cobb angles and (b) compares the SAQ (in its version 1.1) with the TAPS using a set of relevant convergent validity criteria, tests the factor structure, and investigates discriminative validity with a matched healthy control sample. Without such a study, it is impossible to further assess the psychometric quality of both instruments and their applicability in practice and research. As a consequence, the first aim of the present study is to assess the reliability and validity of SAQ and TAPS in detail, based on a large clinical sample and a matched healthy control group. The second aim is to compare both instruments in terms of their quality and provide recommendations for their use in research or by physicians.

## Materials and methods

Patients were recruited from the Department of Orthopaedics at Münster University Hospital in Münster, Germany, and from the self-help group for scoliosis patients in Germany (*Bundesverband Skoliose-Selbsthilfe e.V.*). The online panel PsyWeb (http://psyweb.uni-muenster.de/) was used to establish a healthy control group. Participation in the study required a minimum age of 14 years and was completely voluntary, anonymous, and without any compensation. Informed consent was obtained from all individual participants included in the study. All participants were instructed at the beginning of the online survey about the purpose and responsible researcher (including contact opportunities), that all data will be used only for academic purposes, and that all participants will remain completely anonymous in this study. We asked for consent twice: (1) On the second and third page of the web survey information, consent forms were given. (2) Additionally, all participants were again asked for consent at the end of the study (thus, after they have seen all relevant questions). At this point, participants had the opportunity to withdraw their consent with a self-exclusion item. Data was acquired through self-reports, and data transfer was encrypted. The ethics committee of the Medical Faculty of the University of Münster approved the study (ref. no. 2014-660-f-S).

All study participants were surveyed about their age, gender, height, weight (body mass index was calculated), average level of back pain during the previous 6 months on the visual analogue scale (VAS), current degree of scoliosis (Cobb angle of the most severe curve), history of scoliosis treatment, and current treatment. Afterwards, participants answered several scoliosis-related questionnaires including the SAQ and TAPS. In the SAQ, the first 11 items consist of standardized drawings showing the varying severity of several components of spinal deformity [8]. There are five response options (1–5) with a higher score indicating a more severe deformity. The questionnaire goes on with 22 questions concerning patients’ impressions regarding their appearance with the following answer options (patients choose one): Not true (1), A little true (2), Somewhat true (3), Fairly true (4), and Very true (5). A higher score indicated a worse deformity [8]. The answers to drawings 1 to 10 result in the SAQ Appearance score and questions 12 to 15 in the SAQ Expectations score [8]. Answers to questions/drawings 1 to 10 and 12 to 15 give the SAQ total score (see scoring sheet in Additional file 1: Appendix 1). Sum scores and, for better comparability of the scales, additional mean scores were calculated (Table 1). The TAPS consists of three drawings scored from 1 (greatest deformity) to 5 (smallest deformity), and a mean score is obtained by adding the scores for the three drawings and dividing by 3 (see Additional file 1: Appendix 2). The SAQ Appearance scale and the TAPS are non-verbal. The four verbal items of the SAQ Expectations scale (as well as additional, not analyzed SAQ items and instructions) were systematically translated by a professional medical translator into German and retranslated by a different medical translator into English. Both English versions were compared, and no relevant discrepancies were found. The final German version of the SAQ is attached in Additional file 1: Appendix 1.

**Table 1:** Basic data, demographics, and results of questionnaires in the scoliosis group and control group

Parameter	Scoliosis group (*n* = 255)	Scoliosis subgroup for matched-pair analysis (*n* = 189)	Controls for matched-pair analysis (*n* = 189)
Age (years)	30.0 ± 16.7	33.6 ± 17.0	33.6 ± 17.0
Gender			
Male	38	17	17
Female	217	172	172
Weight (kg)	63.1 ± 11.6	64.1 ± 11.9	67.7 ± 14.4^b^
Height (cm)	169.6 ± 9.5	168.7 ± 9.0	169.1 ± 7.1^c^
BMI (kg/m^2^)	22.0 ± 4.0	22.6 ± 4.2	23.6 ± 4.7^d^
Cobb angle (°)	43.5 ± 20.9	47.8 ± 20.3	–
Academic level			
Secondary school	13	11	2
Junior high	65	47	18
Technical college entry qualification	25	21	10
High school	83	54	67
University degree	69	56	92
Scoliosis treatment^a^			
Physiotherapy	222	160	0
Brace	182	127	0
Surgery	84	73	0
SAQ total score (range 14, i.e., best, to 70, i.e., worst), sum score (mean, range 1, i.e., best, to 5, i.e., worst)	37.29 ± 10.72 (2.87 ± 0.85)	38.58 ± 11.15 (2.94 ± 0.88)	19.19 ± 5.06 (1.45 ± 0.50)^e^
SAQ Appearance (range 10, i.e., best, to 50, i.e., worst), sum score (mean, range 1, i.e., best, to 5, i.e., worst)	23.93 ± 7.59 (2.39 ± 0.76)	25.15 ± 7.83 (2.52 ± 0.78)	12.66 ± 2.49 (1.27 ± 0.25)^e^
SAQ Expectations (range 4, i.e., best, to 20, i.e., worst), sum score (mean, range 1, i.e., best, to 5, i.e., worst)	13.36 ± 4.86 (3.34 ± 1.21)	13.43 ± 4.87 (3.36 ± 1.22)	6.53 ± 3.43 (1.63 ± 0.86)^e^
TAPS (range 5, i.e., best, to 1, i.e., worst)	3.21 ± 0.89	3.07 ± 0.91	4.81 ± 0.31^e^
SRS 22-r score (range 5, i.e., best, to 1, i.e., worst)			
Overall mean	3.76 ± 0.61		
Function	3.89 ± 0.65		
Pain	3.90 ± 0.90		
Self-image	3.46 ± 0.75		
Mental health	3.77 ± 0.78		
Satisfaction	3.77 ± 0.98		
Job-related worries (range 1, i.e., best, to 5, i.e., worst)	2.42 ± 1.24		
Social life-related worries (range 1, i.e., best, to 5, i.e., worst)	2.75 ± 1.29		
Overall stress (range 1, i.e., best, to 5, i.e., worst)	2.37 ± 1.12		
VAS (pain) (range 0, i.e., best, to 10, i.e., worst)	4.20 ± 2.61		
PANAS (mood) (range 10, i.e., best, to 50, i.e., worst)	13.94 ± 5.41		
PHQ-9 (range 0, i.e., best, to 27, i.e., worst)	4.95 ± 4.63		
FKS (body dysmorphic disorder) (range 0, i.e., best, to 64, i.e., worst)	12.17 ± 9.57		
BFI-S (neuroticism) (range 1, i.e., best to 7, i.e., worst)	3.71 ± 1.52		
PTQ (negative thinking) (range 0, i.e., best, to 48, i.e., worst)	18.99 ± 12.43		
WHO-5 (well-being) (range 25, i.e., best, to 0, i.e., worst)	13.50 ± 5.75 (*n* = 133)^f^		

*BMI* body mass index, *SAQ* Spinal Appearance Questionnaire, *TAPS* Trunk Appearance Perception Scale, *SRS* Scoliosis Research Society, *VAS* visual analogue scale, *PANAS* Positive and Negative Affect Schedule, *PHQ-9* Patient Health Questionnaire, *BFI-S* neuroticism subscale of the Big Five Inventory—short, *FKS Fragebogen körperdysmorpher Symptome*, Questionnaire on Body Dysmorphic Symptoms, *PTQ* Perseverative Thinking Questionnaire, *WHO-5* The WHO-5 Well-Being Index

^a^Binomials included

^b^Difference in weight is significant (*T* = − 2.63, df = 376, *P* < 0.01); effect size is rather small (*d* = − 0.27)

^c^Difference in height is not significant (*T* = − 0.50, df = 374, *P* = 0.62)

^d^Difference in BMI is significant (*T* = − 2.24, df = 374, *P* = 0.03); effect size is rather small (*d* = − 0.23)

^e^Differences between scoliosis subgroup and controls are significant with *P* < .01, see the “Discriminate validity” section

^f^Only given during at the second time of measurement (retest)

Furthermore, all participants answered several validated questionnaires including the Scoliosis Research Society 22-r (SRS 22-r) [1], Patient Health Questionnaire (PHQ-9) [13], Positive and Negative Affect Schedule (PANAS; only the negative scale was applied in this study) [14], Questionnaire on Body Dysmorphic Symptoms (*Fragebogen körperdysmorpher Symptome*, FKS) [15], neuroticism subscale from GSOEP Big Five Inventory (BFI-S) [16], and Perseverative Thinking Questionnaire (PTQ) [17]. The study contained three additional questions created by the authors: (1) Do you think your back’s shape will lead to less success in your professional career (job-related worries)? (2) Do you think your back’s shape will lead to less satisfaction in your private life (social life-related worries)? (Answer scale for these two questions is Definitely not (1), Rather not (2), Maybe (3), Probably yes (4), and Definitely yes (5)). 3. All in all, how stressed are you by the look of your back (overall stress)? (Answer scale for this question is Not at all (1), A little bit (2), Moderately (3), Very (4), and Extremely (5)). Due to time restrictions, the WHO-5 Well-Being Index (WHO-5) [18] was only added during a retest measure. At the end of each data collection, participants were thanked, had the opportunity to give additional comments, and could exclude their data from subsequent analysis.

The survey was available online between March 2015 and March 2016. Data were partly used in the validation of the German Body Image Disturbance Questionnaire-Scoliosis (G-BIDQ-S) [19] and the German Quality of Life Profile for Spinal Disorders (G-QLPSD) [20] but were never previously analyzed with respect to the SAQ or TAPS. Further, G-BIDQ-S [19] and the G-QLPSD [20] have a different focus (patients’ specific worries, life quality), following a completely different measurement approach by using verbal items (instead of drawings in SAQ and TAPS), and the main focus of prior publications was an investigation with respect to the success of a German translation; the present paper focuses on psychometric qualities of SAQ and TAPS in general and a recommendation for future application.

Statistical analyses were performed using SPSS, version 23.

## Results

A total number of 677 patients started the questionnaire, yet *n* = 149 dropped out before completing it. Further, we excluded *n* = 181 who reported a spinal deformity other than idiopathic scoliosis, *n* = 87 who reported a Cobb angle below 10°, and *n* = 5 who did not give consent for analyzing their data. Thus, questionnaires of 255 patients (37.67%) were included. An additional 626 individuals were surveyed as healthy controls without scoliosis (of them, *n* = 347 dropped out before completing the questionnaire). This led to a subsample of 189 perfectly matched pairs according to age (full years) and gender (i.e., 74.12% of analyzed patients could be matched).

As the last item of the TAPS showed different drawings for men and women [5], we checked for gender differences in answering behavior before conducting further analysis. In the present study, no significant difference occurred (*M*_men_ = 3.03 ± 1.10 *M*_women_ = 3.14 ± 0.93; *T* = − 0.67, df = 253, *P* = 0.51); thus, item 3 was jointly analyzed for both genders. Basic data, demographics, and the results of the SAQ, TAPS, and the other questionnaires are presented in Table 1.

### Reliability

Reliability was tested in terms of internal consistency (i.e., Cronbach’s *α*) and test–retest reliability (stability over time, see Table 2). Cronbach’s *α* was 0.93 for the SAQ Appearance scale, 0.86 for SAQ Expectations, and 0.91 for SAQ total score; the TAPS had an internal consistency of 0.86. Thus, both measures are highly consistent.

**Table 2:** Reliability of the SAQ and TAPS

	SAQ Appearance	SAQ Expectations	SAQ total score	TAPS	*n*
Cronbach’s *α*	0.93	0.86	0.91	0.86	255
Test–retest reliability	0.84**	0.67**	0.80**	0.84**	133

All patients were invited to the retest, *n* = 133 took part; **P < 0.01

The retest was conducted about 8 weeks after the primary test (on average 55.44 ± 26.32 days). Participants received SAQ and TAPS again, and at both measurement points, some additional measures not pertinent to the current study. There were no significant differences in the means of the SAQ Expectations scale and SAQ total scores. Yet, the SAQ Appearance scale score was a little lower in the retest (T1 25.48 ± 7.87, T2 24.65 ± 8.61; *T* = 2.04, df = 132, *P* = 0.04), and the TAPS score was slightly elevated in the retest (TAPS: T1 3.05 ± 0.93, T2 3.22 ± 0.94; *T* = − 3.82, df = 132, *P* < 0.01). The retest reliability was good (*r* ≥ 0.80, *P* < 0.01) for both measures, except for the SAQ Expectations scale (*r* = 0.67, *P* < 0.01).

### Validity

#### Factorial validity

An exploratory factor analysis (EFA) was used to investigate the structure of both measures. In the analysis of the SAQ, items 1 to 10 and 12 to 15 were included as proposed by Carreon et al. [8]. A value of 0.91 in the Kaiser–Meyer–Olkin (KMO) test indicated high suitability of the data for factor analysis [21]. Screeplot and factor solution reflected exactly the proposed structure of the SAQ with two factors, explaining 58.13% of variance. Factor loadings were between 0.47 and 0.89 for SAQ Appearance items and between 0.70 and 0.81 for SAQ Expectation items (see Additional file 1: Appendix 3). Both scales were correlated (*r* = 0.46, *P* < 0.01, see Additional file 1: Appendix 4).

In the factor analysis of the TAPS, a value of 0.73 in the Kaiser–Meyer–Olkin (KMO) test indicated a middling suitability of the data for factor analysis [21]. The screeplot clearly indicated one single factor, explaining 67.02% of variance. Factor loadings were between 0.77 and 0.85.

In sum, both measures showed clear factor solutions, which indicate high factorial validity.

#### Convergent validity

Convergent validity is the extent of agreement among theoretically highly related measures [22]. The SAQ and its two subscales showed significant correlations with each domain in the SRS 22-r, especially with the SRS self-image scale (see Table 3). Thus, a higher (poorer) SAQ score is associated with a lower (poorer) SRS 22-r score. The same pattern occurred for the TAPS (due to coding, correlations were positive).

**Table 3:** Correlations for convergent, divergent, and concurrent validity

	SAQ Appearance	SAQ Expectations	SAQ total score	TAPS	N
SRS 22-r					
- Overall mean	− 0.49**	− 0.40**	− 0.53**	0.48**	255
- Function	− 0.40**	− 0.20**	− 0.37**	0.40**	255
- Pain	− 0.37**	− 0.24**	− 0.37**	0.36**	255
- Self-image	− 0.53**	− 0.49**	− 0.60**	0.50**	255
- Mental health	− 0.28**	− 0.31**	− 0.34**	0.27**	255
- Satisfaction	− 0.25**	− 0.28**	− 0.31**	0.26**	255
Job-related worries	0.33**	0.31**	0.38**	− 0.33**	255
Social life-related worries	0.26**	0.38**	0.36**	− 0.27**	255
Overall stress	0.51**	0.52**	0.60**	− 0.51**	255
VAS (pain)	0.36**	0.27**	0.38**	− 0.33**	255
Cobb angle	0.55**	0.10	0.44**	− 0.51**	255
BMI	0.41**	0.11	0.34**	− 0.35**	253
PANAS (mood)	0.27**	0.24**	0.30**	− 0.26**	255
PHQ-9 (depression)	0.31**	0.30**	0.35**	− 0.26**	255
FKS (body dysmorphic disorder)	0.26**	0.32**	0.33**	− 0.24**	255
WHO-5 (well-being)	− 0.38**	− 0.24**	− 0.38**	0.33**	133
PTQ (negative thinking)	0.08	0.21**	0.16*	− 0.10	255
BFI-S (neuroticism)	0.16**	0.20**	0.21**	− 0.12*	255

Convergent validation = SRS-22r, job-related worries, social life-related worries, overall stress, VAS, Cobb angle

Divergent validation = BMI

Concurrent validation = PANAS, PHQ-9, FKS, WHO-5, PTQ, BFI-S

*P < 0.05, **P < 0.01

Furthermore, high correlations were found for both measures with overall stress. In addition, worsening SAQ Appearance and TAPS scores were associated with higher Cobb angles, which was further investigated in a subgroup analysis (see below).

#### Divergent validity

Divergent validity refers to the degree of disagreement between theoretically unrelated (or less related) constructs [22]. We expected the SAQ and the TAPS to correlate with the BMI at a low level. Surprisingly, we found relatively high correlations between the BMI and the SAQ Appearance scale (*r* = 0.41), the SAQ total score (*r* = 0.34), and the TAPS (*r* = − 0.35; see Table 3).

#### Concurrent validity

Concurrent validity refers to the ability of a measure to predict a concurrently assessed criterion [22]. The concurrently evaluated criteria (PANAS, PHQ-9, FKS, WHO-5, PTQ, and BFI-S) showed mostly moderate correlations with the SAQ Appearance, Expectations, and total score as well as the TAPS (see Table 3).

#### Discriminant validity

In the context of the present research, discriminant validity refers to the ability of a measure to distinguish between patients with scoliosis and individuals in a healthy control group. In a matched-pair analysis, the scoliosis group and the control group showed very clear differences in both measures: The average SAQ total score was twice as high in patients (see Table 1; *F* = 474.62, df = 1, 376, *P* < 0.01, *d* = 2.24), and the same applied to the SAQ Appearance scale (*F* = 436.72, df = 1, 376, *P* < 0.01, *d* = 2.15) and the SAQ Expectations scale (*F* = 253.69, df = 1, 376, *P* < 0.01, *d* = 1.64). Likewise, the TAPS score was quite lower (i.e., worse) in patients (see Table 1, *T* = − 24.78, df = 231.52, *P* < 0.01, *d* = − 2.56). The effect sizes (*d*)^1^ were very large for all tested differences between patients and controls. Thus, both instruments are highly capable of distinguishing between scoliosis patients and healthy persons.

### Subgroup analysis: Cobb angle and age

A subgroup analysis of patients with Cobb angles of less than 40° and those with ≥ 40° revealed significant differences for SAQ and TAPS, but not for the SAQ Expectations scale (see Table 4). Patients were divided into three age groups (14–17 years, *n* = 59; 18–45 years, *n* = 130; and 46 years and older, *n* = 66). The underage patients group as well as the young adults group showed lower (better) SAQ scores; however, the older patients group showed significantly higher (worse) SAQ scores. Answers given on the TAPS revealed a similar pattern.

**Table 4:** Subgroup and age analysis

	SAQ Appearance	SAQ Expectations	SAQ total score	TAPS
Cobb angle 10 to 39° (*n* = 133)	20.62 ± 4.83	12.89 ± 4.75	33.51 ± 8.22	3.57 ± 0.62
Cobb angle ≥ 40° (*n* = 122)	27.54 ± 8.38	13.88 ± 4.94	41.42 ± 11.61	2.81 ± 0.97
Significance of differences in Cobb angle groups	*F* = 30.26, df = 1, 252, *P* < 0.01	*F* = 1.11, df = 1, 252, *P* = 0.29	*F* = 8.68, df = 1, 252, *P* < 0.01	*F* = 23.11, df = 1, 252, *P* < 0.01
Correlations with age	0.58**	0.18**	0.49**	− 0.59**
Age 14 to < 18 years (*n* = 59)	21.20 ± 5.78	13.92 ± 4.68	35.12 ± 8.55	3.57 ± 0.64
Age 18 to 45 years (*n* = 130)	21.35 ± 5.49	12.18 ± 4.81	33.54 ± 8.93	3.48 ± 0.69
Age ≥ 46 years (*n* = 66)	31.44 ± 7.64	15.20 ± 4.50	46.64 ± 10.24	2.35 ± 0.89
Significance of differences in age groups	*F* = 40.41, df = 1, 252, *P* < 0.01	*F* = 1.16, df = 1, 252, *P* = 0.28	*F* = 10.89, df = 1, 252, *P* < 0.01	*F* = 44.44, df = 1, 252, *P* < 0.01

Means and standard deviations of SAQ sum scores and TAPS mean score are presented; for displayed correlations: **P < 0.01; *F* values result from a MANCOVA with Cobb angle as independent variable, age as covariate, and SAQ and TAPS as dependent variables

## Discussion

In line with prior research, both instruments showed very good results with respect to reliability in terms of Cronbach’s *α* [5, 8, 12]. The TAPS showed good test–retest reliability with a correlation of 0.84. The retest was good for the SAQ Appearance scale (0.84) and the SAQ total score (0.80) but was lower for the SAQ Expectation scale (0.67). Similar values were also reached by Carreon et al. (0.81 for the SAQ Appearance scale and 0.89 for the SAQ total score), but better scores were achieved for the SAQ Expectations scale (0.91) [8]. This difference might be explained by the shorter time period of only 2 weeks between both investigations, in comparison to about 8 weeks after the first interrogation in our study. In light of these results, the long-term stability of the SAQ Expectation scale is at least in doubt and below the requirement (*r* = 0.7) for use in practice [23].

Regarding the convergent validity, the drawings in both questionnaires highly correlate with the Cobb angle (SAQ Appearance: *r* = 0.55, TAPS: *r* = − 0.51), which was also reported in earlier studies and is a lot higher than values found for verbal questionnaires such as the G-BIDQ-S (*r* = 0.30) or G-QLPSD (*r* = 0.28) [12, 19, 20]. The correlation between Cobb angle and both the SAQ Appearance scale and TAPS was even higher than in Carreon et al. (SAQ, *r* = 0.36) and Misterska et al. (TAPS, *r* = − 0.44) [8, 10]. Thus, the use of such drawings might reflect the scoliosis perception of patients much better than verbal questions.

There were also high correlations between the SRS 22-r total score and both questionnaires (SAQ total score *r* = − 0.63 vs. TAPS *r* = 0.48). The highest correlations were reported between the self-image domain of the SRS and the SAQ Appearance scale (*r* = − 0.53) as well as the TAPS (*r* = 0.50). These results match the findings of Carreon et al. (SAQ *r* = − 0.39) and Bago et al. (TAPS *r* = 0.54) [5, 8] and could be explained by the fact that both measures are focused on patients’ body image. Moreover, the overall stress item showed high correlations with both instruments (SAQ, *r* = 0.60 vs. TAPS, *r* = − 0.51), indicating a psychological burden on the patients. The study also revealed evidence for very high discriminant validity: Patients with scoliosis had a significantly higher (worse) SAQ score on both scales as well as for the total score. Similarly, the TAPS score is lower (worse) in patients. This corresponds with earlier findings that patients with scoliosis have a worse body image than healthy controls [19]. Taking everything into account, the SAQ and TAPS showed similar results with regard to various correlations in validation.

The two subgroups of patients with higher and lower Cobb angles (cut-off = 40° according to the international literature, which recommends different treatments for patients below and above 40°) showed similar results on the SAQ Expectations scale. However, patients with more severe deformities had worse scores in the SAQ Appearance scale and the TAPS, which reflects earlier findings in similar studies [10, 12, 19]. Regarding patient age, there seemed to be no relevant difference between underage patients and adults up to the age of 45. However, older scoliosis patients reported worse SAQ and TAPS scores. To date, no studies had been performed concerning this issue; therefore, further research is needed.

With a total number of 255 patients with idiopathic scoliosis, this is the largest collective that has ever answered both the SAQ and the TAPS for the purpose of comparison. Such a sample provides a sound basis for stable estimates of correlations [24]. With regard to factor analysis, most sample size requirements for producing a reliable factor solution were met, although a definitive identification of a multifactorial model might require larger sample sizes [25, 26]. Two further limitations might be considered when interpreting the present study: First, data were acquired via a web-based study relying on self-reports, and no radiographic data for patients were taken into account—as it was most feasible, we only used the main Cobb angle. Second, there might be additional constructs relevant for scoliosis patients’ body image and well-being not covered in the present validation of SAQ and TAPS.

Finally, we aimed to answer the question of which instrument—the SAQ or TAPS—should be recommended for clinical or scientific use. For scientific projects, both could be of value. In clinical everyday situations, the number of questionnaires should be limited due to time restrictions and practicability. Based on the present findings, to investigate patients’ subjective body image, we clearly recommend using the TAPS. Thus, here, we confirm and extend the results of the comparison study performed by Matamalas et al. [12]. The reasons for our recommendation are that, first, the SAQ Appearance scale and TAPS are highly correlated (*r* = 0.85, *P* < 0.01), but the TAPS only consists of three items vs. the ten items on the SAQ’s Appearance scale. Thus, less time is needed to fill out the questionnaire while there is no loss in psychometric quality. Second, as Carreon et al. recommend using only four out of the 22 remaining items of the SAQ [8], using other measures instead of the SAQ Expectations scale seems more efficient and promising. Patient expectations and worries could be better assessed with scales such as the BIDQ-S [2, 19], and scoliosis patients’ quality of life could be better assessed with a measure such as the QLPSD [3, 20].

In general, for treating patients in research, combining different measures is useful in an extensive anamnesis. In doing so, patient questionnaires are of high value for refining a medical diagnosis, understanding a patient’s needs, and assessing the potential need to offer psychotherapeutic support. For clinical use, a compilation of questionnaires is recommended depending on the goals of the caregiver. As a general recommendation, we suggest applying a combination of the TAPS, BIDQ-S, and SRS-22 or alternatively QLPSD as screening instruments for scoliosis patients about twice a year.

## Conclusions

In respect to our first aim, we can state that both instruments show high psychometric qualities; only the stability of the SAQ Expectations scale seems to be impaired. For our second aim, comparing the instruments, we clearly recommend using the TAPS for future clinical workups and research.

## Additional file

Additional file 1: Online supplement. (DOCX 4202 kb)

## Abbreviations

BFI-S: GSOEP Big Five Inventory
BMI: Body mass index
FKS: Fragebogen körperdysmorpher Symptome [Questionnaire on Body Dysmorphic Symptoms]
G-BIDQ-S: German Body Image Disturbance Questionnaire-Scoliosis
G-QLPSD: German Quality of Life Profile for Spinal Disorders
PANAS: Positive and Negative Affect Schedule
PHQ-9: Patient Health Questionnaire
PTQ: Perseverative Thinking Questionnaire
SAQ: Spinal Appearance Questionnaire
SRS 22-r: Scoliosis Research Society 22-r
TAPS: Trunk Appearance Perception Scale
VAS: Visual analogue scale
WRVAS: Walter Reed Visual Assessment Scale

## Footnotes

1 According to the guidelines provided by Cohen, standardized mean differences of 0.2, 0.5, and 0.8 and more are considered to represent small, medium, and large effects, respectively [27].

## References

[1] Niemeyer Thomas, Schubert Christiane, Halm Henry F., Herberts Tina, Leichtle C, Gesicki Marco (2009). Validity and Reliability of an Adapted German Version of Scoliosis Research Society-22 Questionnaire. Spine.

[2] Auerbach Joshua D, Lonner Baron S, Crerand Canice E, Shah Suken A, Flynn John M, Bastrom Tracey, Penn Phedra, Ahn Jennifer, Toombs Courtney, Bharucha Neil, Bowe Whitney P, Newton Peter O (2014). Body Image in Patients with Adolescent Idiopathic Scoliosis. The Journal of Bone and Joint Surgery-American Volume.

[3] Climent José M., Reig Abilio, Sánchez José, Roda Cristina (1995). Construction and Validation of a Specific Quality of Life Instrument for Adolescents With Spine Deformities. Spine.

[4] Sanders James O., Harrast John J., Kuklo Timothy R., Polly David W., Bridwell Keith H., Diab Mohammad, Dormans John P., Drummond Denis S., Emans John B., Johnston Charles E., Lenke Lawrence G., McCarthy Richard E., Newton Peter O., Richards B Stephens, Sucato Daniel J. (2007). The Spinal Appearance Questionnaire. Spine.

[5] Bago J, Sanchez-Raya J, Perez-Grueso FJS, Climent JM (2010). The Trunk Appearance Perception Scale (TAPS): a new tool to evaluate subjective impression of trunk deformity in patients with idiopathic scoliosis. Scoliosis.

[6] Sanders James O., W. Polly David, Cats-Baril William, Jones JoAnn, Lenke Larry G., O’Brien Michael F., Stephens Richards B., Sucato Daniel J. (2003). Analysis of Patient and Parent Assessment of Deformity in Idiopathic Scoliosis Using the Walter Reed Visual Assessment Scale. Spine.

[7] Mulcahey Mary Jane, Chafetz Ross S., Santangelo Anna Marie, Costello Kimberly, Merenda Lisa A., Calhoun Christina, Samdani Amer F., Betz Randal R. (2011). Cognitive Testing of the Spinal Appearance Questionnaire With Typically Developing Youth and Youth With Idiopathic Scoliosis. Journal of Pediatric Orthopaedics.

[8] Carreon LY, Sanders JO, Polly DW, Sucato DJ, Parent S, Roy-Beaudry M, Hopkins J, McClung A, Bratcher KR, Diamond BE (2011). Spinal appearance questionnaire: factor analysis, scoring, reliability, and validity testing. Spine.

[9] Bagó Juan, Climent Jose Ma, Pérez-Grueso Francisco J. S., Pellisé Ferran (2012). Outcome instruments to assess scoliosis surgery. European Spine Journal.

[10] Misterska Ewa, Glowacki Maciej, Latuszewska Joanna, Adamczyk Katarzyna (2012). Perception of stress level, trunk appearance, body function and mental health in females with adolescent idiopathic scoliosis treated conservatively: a longitudinal analysis. Quality of Life Research.

[11] Rigo M, D’agata E, Jelacic M (2012). Trunk Appearance Perception Scale (TAPS) discrepancy between scoliosis children and their parents influence the SRS-22 secore. Scoliosis.

[12] Matamalas A, Bago J, D'Agata E, Pellise F (2014). Body image in idiopathic scoliosis: a comparison study of psychometric properties between four patient-reported outcome instruments. Health Qual Life Outcomes.

[13] Kroenke Kurt, Spitzer Robert L., Williams Janet B. W. (2001). The PHQ-9. Journal of General Internal Medicine.

[14] Watson David, Clark Lee A., Tellegen Auke (1988). Development and validation of brief measures of positive and negative affect: The PANAS scales. Journal of Personality and Social Psychology.

[15] Buhlmann Ulrike, Wilhelm Sabine, Glaesmer Heide, Brähler Elmar, Rief Winfried (2009). Fragebogen körperdysmorpher Symptome (FKS): Ein Screening-Instrument. Verhaltenstherapie.

[16] Schupp J, Gerlitz JY. Big Five Inventory-SOEP (BFI-S). Zusammenstellung sozialwissenschaftlicher Items und Skalen. 2014. 10.6102/zis54.

[17] Ehring Thomas, Zetsche Ulrike, Weidacker Kathrin, Wahl Karina, Schönfeld Sabine, Ehlers Anke (2011). The Perseverative Thinking Questionnaire (PTQ): Validation of a content-independent measure of repetitive negative thinking. Journal of Behavior Therapy and Experimental Psychiatry.

[18] Topp Christian Winther, Østergaard Søren Dinesen, Søndergaard Susan, Bech Per (2015). The WHO-5 Well-Being Index: A Systematic Review of the Literature. Psychotherapy and Psychosomatics.

[19] Wetterkamp Mark, Thielsch Meinald T., Gosheger Georg, Boertz Patrick, Terheyden Jan Henrik, Schulte Tobias L. (2016). German validation of the BIDQ-S questionnaire on body image disturbance in idiopathic scoliosis. European Spine Journal.

[20] Schulte Tobias L., Thielsch Meinald T., Gosheger Georg, Boertz Patrick, Terheyden Jan Henrik, Wetterkamp Mark (2017). German validation of the quality of life profile for spinal disorders (QLPSD). European Spine Journal.

[21] Dziuban Charles D., Shirkey Edwin C. (1974). When is a correlation matrix appropriate for factor analysis? Some decision rules. Psychological Bulletin.

[22] Nunnally JC, Bernstein IH. Psychometric theory. 3rd ed. New York: McGraw-Hill; 1994.

[23] Cook David A., Beckman Thomas J. (2006). Current Concepts in Validity and Reliability for Psychometric Instruments: Theory and Application. The American Journal of Medicine.

[24] Schönbrodt FD, Perugini M. At what sample size do correlations stabilize? J Res Pers. 2013;47:609–12.

[25] Hirschfeld Gerrit, Brachel Ruth von, Thielsch Meinald (2014). Selecting items for Big Five questionnaires: At what sample size do factor loadings stabilize?. Journal of Research in Personality.

[26] Beavers AS, Lounsbury JW, Richards JK, Huck SW, Skolits GJ, Esquivel SL. Practical Considerations for Using Exploratory Factor Analysis in Educational Research. Pract Assess Res Eval. 2013;18(6). Available at: https://www.pareonline.net/getvn.asp?v=18&n=6.

[27] Cohen J (1988). Statistical power analysis for the behavioral sciences.

