# Supplementary material for: Reliability and validity of the Spinal Appearance Questionnaire (SAQ) and the Trunk Appearance Perception Scale (TAPS)
Source: J Orthop Surg Res. 2018 Oct 30;13:274. doi: 10.1186/s13018-018-0980-1 (PMC6208117; doi:10.1186/s13018-018-0980-1)
Supplement: Supplementary file 1 — Online supplement. (DOCX 4202 kb) [file 13018_2018_980_MOESM1_ESM.docx]

*Additional file 1*

Reliability and Validity of the Spinal Appearance Questionnaire (SAQ) and the Trunk Appearance Perception Scale (TAPS)

**Meinald T. Thielsch, Mark Wetterkamp, Patrick Boertz, Georg Gosheger, Tobias L. Schulte**

**Address for correspondence:**

Meinald T. Thielsch, University of Münster, Department of Psychology, Fliednerstr. 21, 48149 Münster, Germany
Email: thielsch@uni-muenster.de
ORCID: orcid.org/0000-0001-8493-9071

# **Appendix 1: German instructions for the SAQ, items and scoring sheet** (drawings are not displayed to avoid copyright conflicts; for original items see Sanders et al., 2007 and Carreon et al., 2011)

**Spinal Appearance Questionnaire (SAQ):**

Im Folgenden wird Ihnen eine Reihe von Bildern präsentiert werden. Bitte wählen Sie jeweils das Bild aus, das Ihrer Wahrnehmung Ihres Körpers am ehesten entspricht.

01. Körperkrümmung (nur eine Markierung)

02. Hervorstehen der Rippen (Buckel)

(nur eine Markierung)

03. Hervorstehen der Flanke (Buckel) (nur eine Markierung)

04. Kopf Brustkorb Hüften (nur eine Markierung)

05. Position des Kopfes über den Hüften

(nur eine Markierung)

06. Schulterstand (nur eine Markierung)

07. Schulterblattrotation (nur eine Markierung)

08. Schulterwinkel (nur eine Markierung)

09. Kopfposition (nur eine Markierung)

10. Hervorstehen der Wirbelsäule (Buckel) (nur eine Markierung)

11. Wählen Sie bitte aus den fünf Bildkategorien diejenige aus,

die Ihnen die meisten Sorgen bereitet.

- Hervorstehen der Rippe
- Hervorstehen der Flanke
- Kopf, Brustkorb, Hüfte
- Schulterstand
- Hervorstehen der Wirbelsäule

Bitte geben Sie an, wie gut die folgenden Aussagen auf Sie zutreffen.

Folgende Antwortmöglichkeiten stehen Ihnen zur Verfügung: Trifft nicht zu - Trifft ein wenig zu – Trifft einigermaßen zu – Trifft ziemlich zu - Trifft sehr zu

12. Ich möchte gerader sein.

13. Ich möchte geradere Schultern haben.

14. Ich möchte geradere Hüften haben.

15. Ich möchte eine gleichmäßigere Taille haben.

16. Ich möchte vorne gleichmäßigere Rippen haben.

17. Ich möchte hinten gleichmäßigere Rippen haben.

18. Ich möchte, dass meine Beinlänge weniger unterschiedlich ist.

19. Ich möchte gleichmäßigere Brüste haben.

20. Ich möchte vorne einen gleichmäßigeren Brustkorb haben.

21. Ich möchte hinten einen gleichmäßigeren Brustkorb haben.

22. Ich möchte in Kleidung besser aussehen.

23. Ich möchte im Badeanzug/ in einer Badehose besser aussehen.

24. Ich möchte in einem Top/Unterhemd besser aussehen.

25. Meine Skoliosenarbe macht mich unsicher (Nur beantworten, wenn Sie wegen Skoliose operiert wurden).

26. Ich möchte attraktiver aussehen.

27. Es gibt andere Dinge an meinem Körper, die mich mehr stören als die Form meines Rückens.

28. Meine Größe stört mich mehr als die Form meines Rückens

29. Mein Gewicht stört mich mehr als die Form meines Rückens

30. Das Aussehen meines Gesichts stört mich mehr als die Form meines Rückens.

31. Welche der Fragen 12–30 ist für Sie am wichtigsten?

32. Wie würden Sie Ihr Selbstbild bewerten? Hiermit ist gemeint, wie Sie über sich selbst und Ihren Körper denken.

Antworten: Sehr schlecht - Schlecht - Mittelmäßig - Gut - Sehr gut

33. Was möchten Sie an der Form Ihres Körpers am liebsten verändern und warum? (Freitextantwort)

*Scoring sheet for the SAQ:*

|  | **Sum score** | **Mean score** |
| --- | --- | --- |
| SAQ Appearance (Questions 1-10)  (scores from 1 to 5) | 1 + 2 + 3 + 4 + 5 + 6 + 7 + 8 + 9 + 10 | (1 + 2 + 3 + 4 + 5 + 6 + 7 + 8 + 9 + 10) / 10 |
| SAQ Expectations (Questions 12-15) (scores from 1 to 5) | 12 + 13 +14 +15 | (12 + 13 +14 +15) / 4 |
| SAQ total score (Questions 1-10 and 12-15) | 1 + 2 + 3 + 4 + 5 + 6 + 7 + 8 + 9 + 10 + 12 + 13 +14 +15 | (1 + 2 + 3 + 4 + 5 + 6 + 7 + 8 + 9 + 10 + 12 + 13 +14 +15) / 14 |

Note. There were five response options (1-5). For questions 1-10, there were five different drawings to choose from (scores 1 to 5). Questions 12-15 could be answered with one of the following options: Not true (1) – A little true (2) – Somewhat true (3) – Fairly true (4) - Very true (5). A higher score indicated a worse deformity. Scores for SAQ Appearance and SAQ Expectations scales are added up to build a sum score. Nevertheless, for better comparability of scales, mean scores should be calculated as well.

**Appendix 2: TAPS with German instruction, items and scoring sheet** (for original items see Bago et al., 2010)

**Trunk Appearance Perception Scale (TAPS):**

Bitte sehen Sie sich die folgenden Abbildungen mit verschieden geformten Wirbelsäulen genau an. Klicken Sie in jeder Abbildung eine Skizze an, die Ihnen am stärksten ähnelt.

*Scoring sheet for the TAPS:*

|  | **Mean score** |
| --- | --- |
| TAPS total score | (1 + 2 + 3) / 3 |

Note. The TAPS consists of three drawings scored from 1 (greatest deformity) to 5 (smallest deformity), and a mean score is obtained by adding the scores for the 3 drawings and dividing by 3. For example, for the drawing on the very left the score is 5, and for the drawing on the very right the score is 1.

# **Appendix 3: Factor loadings for SAQ items**

|  | | | |
| --- | --- | --- | --- |
|  | Factor | |  |
|  | 1 | 2 |  |
| 1. Body curve | 0.804 |  |  |
| 2. Rip prominence (bump) | 0.842 |  |  |
| 3. Flank prominence (bump) | 0.583 |  |  |
| 4. Head chest hips | 0.799 |  |  |
| 5. Position of head of hips | 0.467 |  |  |
| 6. Shoulder level | 0.613 |  |  |
| 7. Shoulder blade rotation | 0.889 |  |  |
| 8. Shoulder angle | 0.874 |  |  |
| 9. Head position | 0.610 |  |  |
| 10. Spine prominence (bump) | 0.802 |  |  |
| 12. I want to be more even (German: Ich möchte gerader sein) |  | 0.695 |  |
| 13. I want to have more even shoulders (German: Ich möchte geradere Schultern haben |  | 0.766 |  |
| 14. I want to have more even hips (German: Ich möchte geradere Hüften haben) |  | 0.807 |  |
| 15. I want to have a more even waist (German: Ich möchte eine gleichmäßigere Taille haben) |  | 0.813 |  |

Note: Factor 1 reflects the SAQ Appearance scale, factor 2 the SAQ Expectations scale. Loadings < 0.3 are not displayed.

# **Appendix 4: Intercorrelations of SAQ subscales, SAQ total score and TAPS**

|  | SAQ Expectations | SAQ total score | TAPS |
| --- | --- | --- | --- |
| SAQ Appearance | 0.458** | 0.773** | -0.849** |
| SAQ Expectations |  | 0.918** | -0.415** |
| SAQ total score |  |  | -0.674** |

Note: **p <0.01.
